# COVID-19 on Oral Health: A New Bilateral Connection for the Pandemic

**DOI:** 10.3390/biomedicines12010060

**Published:** 2023-12-26

**Authors:** Luigi Bellocchio, Gianna Dipalma, Angelo Michele Inchingolo, Alessio Danilo Inchingolo, Laura Ferrante, Gaetano Del Vecchio, Giuseppina Malcangi, Andrea Palermo, Andis Qendro, Francesco Inchingolo

**Affiliations:** 1 INSERM, U1215 NeuroCentre Magendie, Endocannabinoids and Neuroadaptation, University of Bordeaux, 33063 Bordeaux, France; luigi.bellocchio@inserm.fr; 2 Department of Interdisciplinary Medicine, University of Study “Aldo Moro”, 70124 Bari, Italy; angeloinchingolo@gmail.com (A.M.I.); ad.inchingolo@libero.it (A.D.I.); lauraferrante79@virgilio.it (L.F.); dr.gdelvecchio@gmail.com (G.D.V.); francesco.inchingolo@uniba.it (F.I.); 3 College of Medicine and Dentistry, Birmingham B4 6BN, UK; andrea.palermo2004@libero.it; 4 Faculty of Dental Medicine, University of Medicine, 1005 Tirana, Albania; andis.qendro@umed.edu.al

**Keywords:** COVID-19, SARS-CoV-2, saliva transmission, respiratory syndrome, infection, oral cavity (O.C.), virus signs and symptoms

## Abstract

Severe acute respiratory syndrome coronavirus 2 (SARS-CoV-2) infection and transmission are generally known to be produced by respiratory droplets and aerosols from the oral cavity (O.C.) of infected subjects, as stated by the World Health Organization. Saliva also retains the viral particles and aids in the spread of COVID-19. Angiotensin-converting enzyme Type 2 (ACE2) and transmembrane serine protease 2 (TMPRSS2) are two of the numerous factors that promote SARS-CoV-2 infection, expressed by O.C. structures, various mucosa types, and the epithelia of salivary glands. A systemic SARS-CoV-2 infection might result from viral replication in O.C. cells. On the other hand, cellular damage of different subtypes in the O.C. might be associated with various clinical signs and symptoms. Factors interfering with SARS-CoV-2 infection potential might represent fertile ground for possible local pharmacotherapeutic interventions, which may confine SARS-CoV-2 virus entry and transmission in the O.C., finally representing a way to reduce COVID-19 incidence and severity.

## 1. Introduction

**Figure 1:**
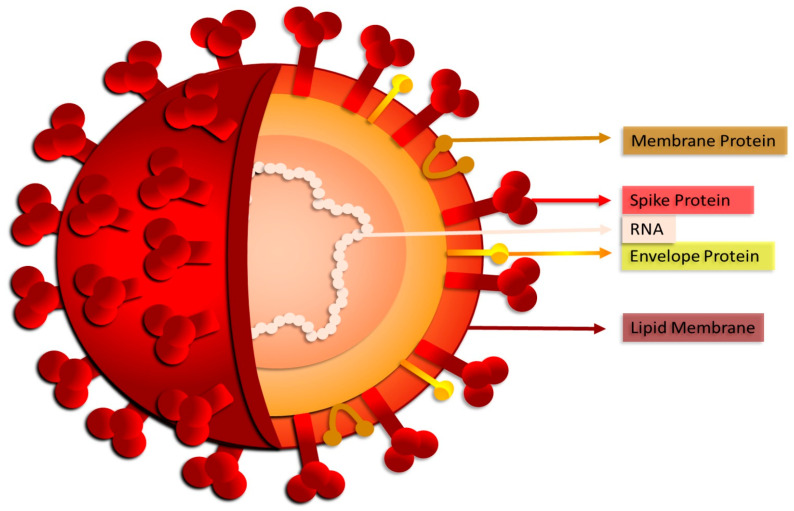
SARS-CoV-2: structure of the new coronavirus.

In 2019, a new type of coronavirus, SARS-CoV-2 (Figure 1), the ethiopathological agent of COVID-19, was detected in Wuhan (China), and on 11 March 2020, it became a pandemic, according to the World Health Organization.

**Figure 2:**
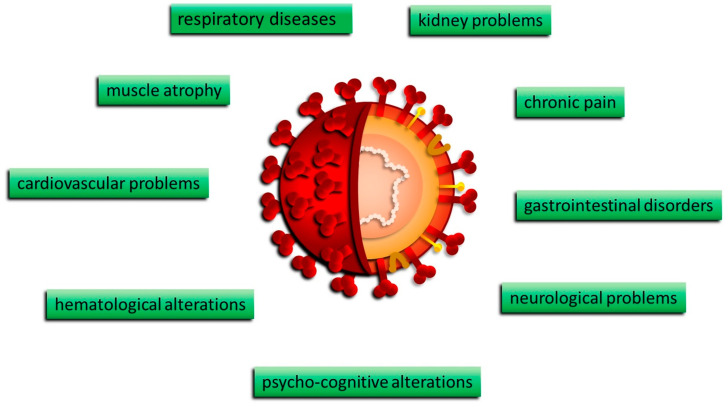
Clinical manifestations of COVID-19.

The manifestations of COVID-19 were mostly age-dependent, related to the clinical stage of the infection, and potentially more severe in cases already presenting co-morbidities. COVID-19 disease is characterized by runny nose and nasal congestion, anosmia, dysgeusia or hypogeusia, diarrhea, nausea/vomiting, respiratory distress, fatigue, ocular symptoms, diarrhea, vomiting, and abdominal pain. These systemic conditions were often accompanied by skin and mucosal lesions (Figure 2). 

Several oral lesions were found in COVID-19 patients. Amongst them, the most commonly reported are: geographic tongue, herpes simplex, aphthous-like ulcers, candidiasis, hemorrhagic and necrotic ulcerations, erythematous surfaces, reddish macules, white hairy tongue, petechiae, and pustular enanthema [1,2]. This review article will consider and analyze the existing evidence on the molecular processes of O.C. infection by SARS-CoV-2. In half of COVID-19 patients, viral infection was associated with taste loss, dry mouth, and mucosal lesions. Indeed, recent findings point out that the O.C. is not only the primary site of SARS-CoV-2 entrance and transmission but also a target for the disease’s clinical presentation [3,4]. The implication of oral tissue in COVID-19 pathogenesis is supported by growing evidence, which confirms the hypothesis of direct viral entry and replication of mucosal surfaces and the salivary glands [5,6,7].

**Figure 3:**
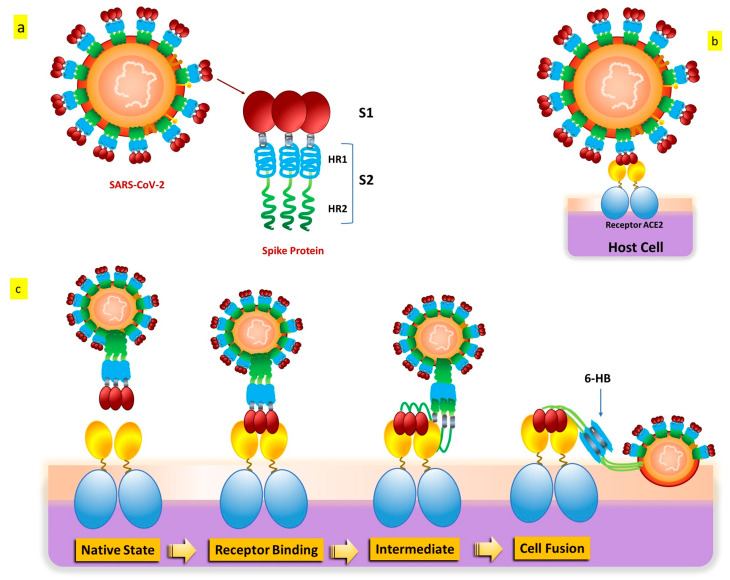
SARS-CoV-2 S protein: (**a**) The S protein’s schematic structure. (**b**) The S protein attaches itself to the ACE2 receptor. (**c**) The S protein-mediated binding and virus-cell fusion mechanisms.

SARS-CoV-2 penetrates cells in two different ways, such as endocytosis or host membrane-bound peptidases [8,9]. SARS-CoV-2 can start its viral envelope entrance by attaching its spike protein (S) to the metallopeptidase angiotensin-converting enzyme 2 (ACE2) that is present on the cellular membrane [10,11]. A protease derived from a host cell subsequently divides the spike into S1 and S2, respectively. S1 separates from the remainder related to the spike protein, and host cell-derived transmembrane serine protease 2 (TMPRSS2) further cleaves S2 (Figure 3) [12].

This cleavage process results in exposure to the fusion peptide, which allows fusion of the membrane of the host cell as well as membrane and cell invasion [13]. Some in vitro evidence indicates that another entry factor that potentiates SARS-CoV-2’s infectivity [14] is represented by neuropilin-1 (NRP-1), a signaling protein highly present in the O.C. In addition to the well-known roles played by ACE2 and TMPRSS2, additional endosomal proteases (CTSB, CTSL, and BSG) and tissue-specific proteases (TMPRSS4 and TMPRSS11D) might facilitate the virus’s entrance into cells for intracellular reproduction [15,16]. Interaction of the cell membrane-based receptor ACE2 of the host cell with the spike protein of SARS-CoV-2 triggers the endosomal pathway internalization, which results in virus endocytosis [13,17]. After that, the endosome’s cathepsin L cleaves the spike protein into S1 and S2, allowing the viral capsid to merge with the membrane within the endosome [18,19,20]. The virus genome is therefore released because of endosomal processing, allowing it to begin replicating and producing new viral particles. Therefore, it appears that ACE2 binding and TMPRSS2 cleavage are the two primary crucial components in the SARS-CoV-2 infection process, even if several pathways and intracellular players are involved [21,22].

## 2. SARS-CoV-2 Transmission via the Saliva

**Figure 4:**
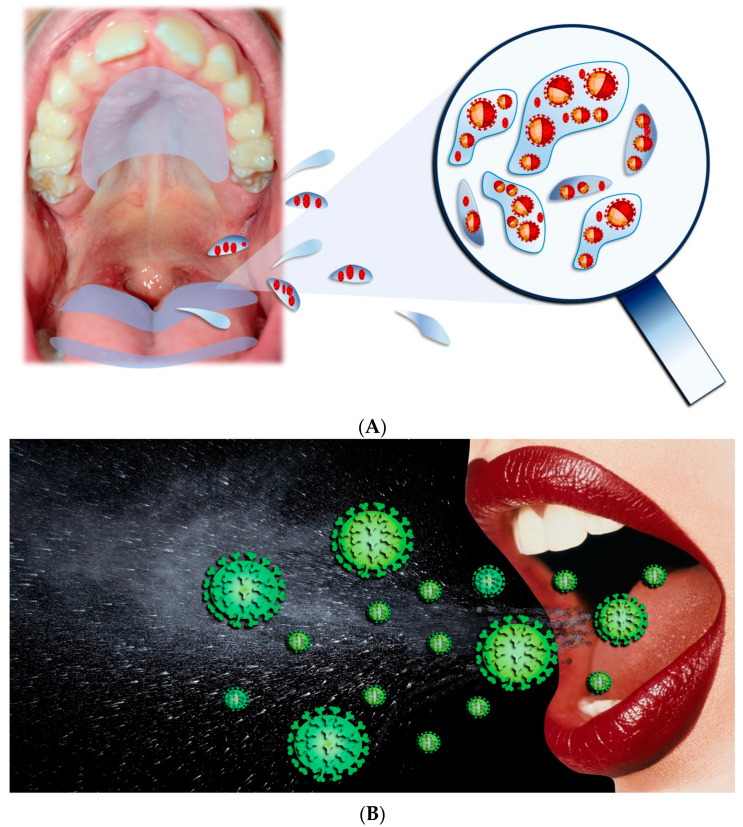
(**A**,**B**) Respiratory droplets originated from the O.C.

The main paths for transmission for SARS-CoV-2 are Respiratory droplets, Flügge (Figure 4), which originate from the nose, O.C., and airways [23,24,25,26], making saliva the most significant droplet [27,28,29]. In the O.C., SARS-CoV-2 viral RNA titers range generally from 102 to 1010 copies/mL, reaching, in the first seven days, the highest concentrations of symptom appearance and declining with recovery over time [30,31].

In saliva, fragments of oral mucosa, as well as salivary gland cells, are normally and mutually present. Indeed, in saliva samples obtained from light COVID-19 people, SARS-CoV-2 was reported to infect about 5–10% of salivary cells (pan-cytokeratin positive, pCK+) [27]. The observation that in lost cells in the salivary epithelium viral replication is happening is increasing the potential of saliva in the spread of infection and disease transmission. Also, suprabasal mucosal cells express all factors required for both SARS-CoV-2 entry and replication. These cells, which are normally shed as a potential protective phenomenon in oral tissue infection [32], are generated from the layers of tissue that are most terminally differentiated every 3 h and can represent a way SARS-CoV-2 is applicable to spread into saliva [8]. In vitro studies supported this hypothesis, showing that these cells were able to transmit a high viral load [27,33] from saliva to Vero cells in an ex vivo experimental setup. Moreover, a cell population normally present within the respiratory tract (i.e., pCK+ ciliated cells) was identified in the saliva while being positive for SARS-CoV-2. Thus SARS-CoV2 infection and propagation in the O.C. [34,35] with subsequent sustained COVID-19 in different body sites might be partly due to the high saliva viral load after the spreading of the cell population within the respiratory tract in this body fluid [34]. Furthermore, the lower respiratory tract being infected as the digestive apparatus might involve exfoliated epithelial cells present in saliva, containing active SARS-CoV-2 particles [36,37].

Refs. [27,38,39] a stable capacity for infection in fresh cell monolayers was also attributed to viral particles from culture supernatants with a cytopathic effect [40]. Altogether, these findings further strengthen the idea of saliva’s capacity to spread SARS-CoV-2, as pathogenic virus and diseased cells found in oral droplets that were ejected, including individuals without apparent symptoms or those in the early stages of the infection, represent the origin of airborne transmission [27,41,42]. SARS-CoV-2 can be detected in saliva for extended periods of time; for example, in asymptomatic subjects, weeks pass between the initial test and the negative saliva test result, a delay that is even longer in symptomatic COVID-19 patients [27,43]. The virus SARS-CoV-2 excludes the ability to be removed from the nasopharynx in saliva, as suggested by other observations, and this might indicate prolonged viral shedding from oral areas affected by SARS-CoV-2 [27,44,45].

Also, the periodontium has been cited as a possible site for SARS-CoV-2 replication and subsequent release. From this tissue, saliva and O.C. are highly accessible to the virus [46]. However, it can potentially disseminate to other distant organs by entering the local periodontal capillary network’s circulation. Thus, the mouth cavity is a key node not only as a potential external source of infection but also for the recurrence and development of systemic COVID-19 disease [47,48,49]. A recent postmortem investigation discovered the presence of SARS-CoV-2 RNA in the periodontal tissues of COVID-19-positive patients, perhaps indicating the virus’s existence within crevicular fluid several days after the beginning of the first symptoms [50]. Therefore, the periodontal pocket may serve as an advantageous reservoir for latent and active SARS-CoV-2 isoforms [5,47]. As a result, the mouth cavity is a crucial location and a possible pathway for the cellular and acellular particles of SARS-CoV-2 to become infected through saliva [2,27].

In addition to the reasons listed above that make saliva dangerous for the general public, other infectious material can expose dental practitioners during routine procedures in O.C. Components facilitating the entry and spread of SARS-CoV-2 were identified in dental pulp tissues, and lesions around the tooth root [27,51,52] suggest the possibility of the virus colonizing pulp tissues during pathological conditions such as caries or through a bloodstream-related infection in the pulp [53]. Dental and periodontal procedures may pose transmission risks of SARS-CoV-2 to dental professionals. Therefore, implementing specific preventive measures is crucial to mitigating the transmission of the disease during O.C.-related interventions [54,55]. We can therefore underline how much connection there is between SARS-CoV-2 infection and oral health. The O.C. stands as one of the initial points of entry for the virus; viral particles present in saliva can be widespread through direct contact or sharing contaminated objects. This has important implications for the propagation of the virus inside communities and highlights the importance of oral hygiene. People with pre-existing oral conditions, such as periodontitis or other gum disease, may be at potentially greater risk of becoming infected with COVID-19 or developing more severe forms of the disease. This may be attributable to local immune system impairment in inflamed areas of the mouth, making the body more susceptible to SARS-CoV-2 infection. The pandemic has led to changes in dental practices, including the adoption of more rigorous protocols for infection prevention. This included the use of protective equipment, changes in procedures to minimize the production of aerosols, and the implementation of social distancing measures in dental centers.

The connection between COVID-19 and oral health has been the subject of ongoing study to better understand the impact of the infection on the mouth and vice versa. Continuing to follow health guidelines, including good oral hygiene, is important not only for preventing COVID-19 infection but also for maintaining overall good oral and dental health [56,57].

## 3. SARS-CoV-2 and Oral Cavity: A New Entry Route in the Body

### 3.1. Salivary Glands

One major entry point for the infection of SARS-CoV-2 appears to be the epithelia of the glands that produce saliva. Here, virus entry factors are expressed at very high levels compared to other O.C. epithelial cells. The SARS-CoV-2 virus can infect the upper airways, including the mouth, in several ways:

1. The virus can enter the O.C. through the upper respiratory tract, such as the nose and throat, mainly via respiratory droplets released when an infected person coughs, sneezes, or talks. Viral particles can be inhaled or deposited on the surfaces of the mouth and nose.
2. Once in the O.C., the SARS-CoV-2 virus binds to ACE2 receptors present on host cells. This is the entry point of the virus into human cells.
3. The virus can penetrate the oral mucosa through adhesion and invasion of the epithelial cells present in this region. This process could be favored by lesions or microlesions in the mucosa, providing an entry route for the virus.
4. Once inside the oral mucosa, the virus can be transmitted to the salivary glands via the lymphatic or circulatory system. In saliva, the existence of SARS-CoV-2 has been observed, suggesting that the virus may be transported through the saliva itself or through the bloodstream.

ACE2 and TMPRSS2 immunoreactivity was found both in mucous and serous sacs within the labial gland, with stronger TMPRSS2 levels in the acini; moreover, ACE2 was detected in the striated ducts; however, TMPRSS2 staining seemed to show a negative result [58,59]. Thus, SARS-CoV-2 might primarily attach to the mucosa of O.C., the ductal opening of the salivary glands, and the small salivary glands scattered throughout the oral mucosa [58]. Zhu and colleagues analyzed the location of SARS-CoV-2 entry points, offering insights into the primary salivary glands of individuals with non-malignant conditions [60]. Specifically, within the submandibular and parotid glands, ACE2 and TMPRSS2 proteins were detected in the cytoplasm and cellular membrane of serous acinar cells, ductal epithelial cells, and mixed acini’s serous acinar cells within the sublingual glands [6,61]. Lower levels of ACE2 and TMPRSS2 were found in the submandibular, parotid, and sublingual glands, respectively, with Western blot analysis for protein quantification (particularly for TMPRSS2) [60,62].

Single-cell RNA sequencing revealed co-expression of ACE2 and protease TMPRSS2 (together with additional proteases CTSB and CTSL) in the salivary gland epithelial cells (parotid, labial minor, and submandibular), and co-in situ hybridization investigations confirmed these findings. It is interesting to note that the authors discovered distinct tissue-specific protease expression patterns, with TMPRSS2 abundant in the epithelia of salivary glands and TMPRSS11D rich in mucosal keratinocytes [63]. These differential patterns of protease expression might be indicative of tissue-specific infection routes [27,64,65]. On the other hand, the endosomal proteases CTSB and CTSL showed broader expression levels across epithelia [66,67]. Indeed, the entry factors were more highly expressed in the salivary glands (especially the minor ones) than in the O.C. mucosa; importantly, significant co-expression of the principal entry factors ACE2 and TMPRSS2 was found in acini and duct epithelial cells [58,68,69]. The levels of these factors in the minor salivary glands were comparable to those found in the respiratory tract and gastrointestinal tract [27,70,71]. In salivary gland homogenates, SARS-CoV-2 spike proteins were able to attach to human parotid, submandibular, and sublingual gland cells [60], confirming their infectious potential at this anatomical site [62,72,73]. Like the observations for the oral mucosa, Huang and colleagues reported SARS-CoV-2 replication in salivary glands. Indeed, using minor salivary glands from corpses and a person who was severely infected with COVID-19, the authors reported the presence of replicated viral particles in infected ducts and acini and a lower infection in parotid salivary glands [60,74]. Submandibular gland infection by SARS-CoV-2 was reported in two different studies [50,75,76]. In 60% of submandibular and parotid gland specimens, an electron microscopy study of postmortem biopsies of fatal COVID-19 patients revealed spherical 70–100 nm virus particles positive for SARS-CoV-2 RNA (consistent in size and shape with the Coronaviridae family) [50,77]. However, it is important to underline that scientific research in this field is continually developing, and there are still many questions without definitive answers. A complete understanding of the specific mechanisms by which the virus infects the O.C. and salivary glands requires further studies and insights.

It is always advisable to follow recommended health guidelines, such as hand hygiene, wearing masks, and social distancing, to reduce the risk of contracting or spreading the virus.

### 3.2. Tongue

Gustatory dysfunction affected around 40% of COVID-19 patients, and it mostly manifested two to fourteen days after exposure to SARS-CoV-2 [5,6]. This can be explained by the SARS-CoV-2 invasion of entry taste papillae cells, resulting in cellular harm and leading to the clinical symptom of dysgeusia [78]. In the mucous membrane of the tongue, immunohistochemistry studies showed ACE2 expression within the cell cytoplasm and across the cellular membrane within the non-keratinized area, alongside TMPRSS2 positioning specifically on the cellular membrane [79,80]. ACE2 was detected in minor quantities within the lamina propria of non-keratinized stratified squamous epithelia in mucosal structures. Conversely, TMPRSS2 expression was absent in the stratum basale or within the lamina propria [58,81,82]. Extended immunohistochemical examination revealed ACE2 presence within the nucleus and TMPRSS2 within the cytoplasm of taste cells in the papillae. These findings were additionally corroborated by RNA analysis conducted on human fungiform papillae taste cells [82]. Xu and colleagues further confirm ACE2 receptor presence in the tongue (especially abundant in epithelial cells) by using single-cell sequencing [83,84].

As already mentioned for the oral mucosa, the existence of SARS-CoV-2 entry components within the tongue might represent another infection gateway. Indeed, in SARS-CoV-2-infected subjects as well as autopsy patients, viral infection was found in the dorsal tongue [27,85,86]. Seventy-one percent of COVID-19 patients exhibited cytological smears from the tongue’s dorsum, with epithelial cells testing positive for the SARS-CoV-2 spike protein [87,88].

### 3.3. Oral Mucosa

Expression of TMPRSS and ACE2, together with other cellular factors for SARS-CoV-2 infection, has been identified in the tongue, oral mucosa, and salivary glands [81,89,90]. Immunohistochemistry studies showed ACE2 expression in the cytoplasm and on the cell membrane in the non-keratinized buccal mucosa, as well as TMPRSS2 localization on the cell membrane [91]. ACE2 was detected in both the cytoplasm and on the cell membrane, while TMPRSS2 was found solely on the cell membrane within the non-keratinized stratified squamous epithelia of the labial mucosa. Conversely, within the buccal mucosa, significant ACE2 levels were observed in the lamina propria, with no expression of TMPRSS2 noted in either the stratum basale or the lamina propria [58,92]. ACE2 was observed within the cytoplasm and on the cell membrane, while TMPRSS2 was specifically identified on the cell membrane. These were detected in the keratinized stratified squamous epithelia, primarily localized in the stratum granulosum and stratum spinosum, with no presence noted in the stratum basale [56]. Another study corroborated these findings, identifying immunoreactivity of both ACE2 and TMPRSS2 within the stratified squamous epithelium of the gingiva, specifically prevalent in the keratinized surface layer [85]. In the same sites, this study also showed the positioning of furin [82,93,94]. Furin is the second protease, in addition to TMPRSS2, used by SARS-CoV-2 to cleave the Spike protein that anchors to the cell membrane, without whose cleavage the cellular entry of the virus would not take place and therefore neither would replication and infection [95,96,97]. Another study by Okui and colleagues, utilizing gingival cells obtained from the gingival sulcus, demonstrated ACE2 immunoreactivity comparable to levels found in the tongue. Historically considered one of the primary entry routes for SARS-CoV-2, alongside the salivary glands, the gingiva showed consistent ACE2 expression in this study conducted by colleagues [36,98,99,100]. In the same study, low ACE2 expression levels were also found within the keratinized mucosa in the palate [100,101,102]. Through single-cell RNA sequencing analysis, SARS-CoV-2 entry factors were identified across various subtypes of oral epithelial cells. Specifically, mucosal keratinocytes were found to express ACE2, TMPRSS2, as well as the endosomal proteases CTSB and CTSL [21,27,103]. Xu and colleagues provided further single-cell sequencing data showing ACE2 receptor expression within the tissue of the inner cheek and the gums [83,104]. Interestingly, the co-expression levels of ACE2 and TMPRSS2 in the oral mucosa were found to be similar to those observed in nasal and intestinal epithelial cells [105,106,107], the best known sites of SARS-CoV-2 infection [108,109]. In healthy adult tissue samples of the inner cheek, both suprabasal and basal compartments (non-keratinized) displayed ACE2 and TMPRSS2 expression, identified through in situ hybridization. Similar findings were observed in the soft palate and palatine tissue [40,83,110].

Thus, multiple regions of the O.C. are potential targets for SARS-CoV-2 infection, carrying the potential for viral transmission to both the respiratory and gastrointestinal tracts [100]. ACE2 and TMPRSS2 presence in the periodontal pocket and sulcular epithelia, coupled to the crevices, gingival sulcus, and periodontal pocket microenvironment, provide all conditions conducive to virus replication and sustainability [91,111].

Huang and colleagues clearly demonstrated the presence of SARS-CoV-2 colonization within the oral mucosa [27,112]. Spike protein was detected independently within shed epithelial cells and on their membrane surface. Infection and replication of SARS-CoV-2 were observed across all layers of the mucosa, with signs of infection also detected in mucosal scrapings [113].

### 3.4. Dental Pulp

SARS-CoV-2 entry components, namely ACE2 and TMPRSS2, exhibit substantial expression in both healthy and inflamed human dental pulp, as revealed by comprehensive transcriptomic analysis [114]. Accordingly, another study confirmed these findings by demonstrating RNA expression of ACE2, TMPRSS2, and NRP1 within healthy pulp tissues [52], indicating that SARS-CoV-2 infection can occur in pulp regardless of inflammatory status [115,116].

## 4. SARS-CoV-2 Infection of O.C. Is Controlled by Different Factors

The expression of virus entry factors is dependent on both age and sex, as pointed out by several clinical observations. Elderly women and men exhibit relatively higher mRNA expression levels of ACE2 and TMPRSS2 in the oral mucosa compared to younger individuals, regardless of gender [117,118]. No sex differences were found for ACE2 expression in oral tissues; on the other hand, mRNA expression of TMPRSS2 was observed to be lower in the oral tissues of females [117,118,119]. Confirmation via Western blotting and immunohistochemistry validated the RNA expression findings, indicating notably elevated ACE2 and TMPRSS2 protein levels in the mucosa of elderly subjects compared to younger ones. Interestingly, at a comparable age, no significant differences in protein expression were observed between females and males. The heightened levels of ACE2 and TMPRSS2 in older males and cohorts correlated positively with an increased SARS-CoV-2 presence in saliva [117]. According to these observations, oral SARS-CoV-2 infection might occur preferentially in the elderly population, and, in this scenario, clinical observations reported predominant clinical symptoms and infection in elderly populations [32]. Cytokines, proteins produced by immune system cells, regulate inflammatory responses in the body. In the context of periodontitis and COVID-19, there has been growing interest regarding the role of periodontal cytokines and bacteria in the interaction with the SARS-CoV-2 virus. Some cytokines produced in response to periodontal bacterial infection could amplify the inflammatory response in the body, potentially increasing the susceptibility or severity of COVID-19 infection. This may be due to an interaction between the local immune response in the mouth and the systemic response to the virus. However, understanding the specific details of this process requires further study. Impact of COVID-19 on periodontitis and the oral microbiome COVID-19 has been shown to impact oral health in several ways:

- Aggravation of periodontitis: Systemic inflammation caused by the COVID-19 infection could affect oral health conditions, including periodontitis. This inflammatory condition of the gums may be exacerbated or worsened due to systemic stress and an altered immune response during the COVID-19 infection.
- Changes in the oral microbiome: some studies have suggested that prolonged use of medical devices such as ventilators or the effect of drugs used to treat COVID-19 could alter the oral microbiome. This could affect the balance of bacteria in your mouth, potentially increasing your risk of developing disease conditions such as periodontitis.

SARS-CoV-2 entry can also be modulated by local inflammatory processes in the O.C. The pathogenesis of periodontal diseases, such as *Porphyromonas gingivalis* and cytokines, can potentially influence the expression of molecules involved in SARS-CoV-2 entry and processing, at least in vitro. For instance, *Porphyromonas gingivalis*-derived lipopolysaccharide (PgLPS), IL1β, TNFα, and PGE2 were observed to notably elevate ACE2 and TMPRSS2 expression while reducing furin expression [120], indicating that inflammation localized in the periodontal gingiva may favor virus infection. The lytic activity of periodontal bacteria can synergistically act with membrane proteases, prompting early and sustained colonization of the O.C. by SARS-CoV-2 [50,121], as evidenced by the detection of the virus in saliva samples prior to the onset of clinical symptoms [122] lingering for a duration even after the relief of symptoms [123]. Similarly, a case-control study established a strong correlation between periodontitis and the severity of COVID-19 infection [47,124,125,126]. Indeed, this report revealed that an increased risk of ICU admission, the need for assisted ventilation, and mortality among COVID-19 patients was accompanied by periodontitis [84].

The inflammatory reaction in periapical tissues, including the action of IL-6, appears to differ from the aforementioned periodontal tissues. The expression of ACE2, TMPRSS2, and NRP-1 was notably decreased in oral periapical lesions (such as periapical abscesses, preapical granulomas, and radicular cysts) when compared to healthy pulp tissues [82], and in periapical abscesses and granulomas, a negative correlation between IL6 and the gene expression of ACE2, NRP1, and TMPRSS2 was documented. However, another study failed to identify variations in the expression levels of both ACE2 and TMPRSS2 in samples obtained from healthy and inflamed dental pulp. These findings recapitulate the expression patterns found in other tissues [58,127], thus suggesting that inflammatory conditions do not change ACE2 and TMPRSS2 expression [128].

Increase in SARS-CoV-2 Another component in saliva that might alter the risk of contracting SARS-CoV-2 infection is 1 cross-reactive IgA. In fact, IgA inhibited the SARS-CoV-2 spike protein’s ability to bind to ACE2 receptors to some extent. This antibody was found in almost half of those who never had an infection with SARS-CoV-2 [81]. These antibodies do, in fact, correspond to cross-reacting antibodies of other homologous coronaviruses, suggesting that the salivary presence of these antibodies (particularly IgA, which typically declines with age) may help prevent infection with SARS-CoV-2. Therefore, lower levels in older participants may be the reason for the increased viral contagious potential and COVID-19 incidence [81,129]. Therefore, IgA protective activity may account for the clinical results of a less severe (and commonly asymptomatic) COVID-19 course in children and adolescents [64,130,131]. The early specific humoral response constituted by IgA antibodies against SARS-CoV-2 was also detected and persisted in saliva in COVID-19 patients [132,133]. Consequently, these antibodies can be viewed as a particular defensive mechanism and may one day be investigated as a possible diagnostic parameter [134,135,136].

In addition, age immunity and inflammatory status, SARS-CoV-2 infection in O.C. might also be modulated by environmental factors [38,137,138]. Cigarette smoke condensates induce the expression of ACE2 and TMPRSS2 in human gingival epithelial cells, suggesting that smoking can enhance one’s vulnerability to COVID-19 illness [139,140,141,142]. The internalization of a SARS-CoV-2 pseudovirus in the cells was enhanced in the same research by exposure to cigarette smoke condensates in an AhR-dependent way (which is a nuclear receptor known to mediate cigarette smoke responses) [6,40,143]. Thus, these experimental findings suggest the possibility of reduced coronavirus infection of the O.C. structures by smoking cessation [144,145]. The SARS-CoV-2 virus itself has the ability to modify the expression of ACE2 in the oral mucosa, as evidenced by the downregulation of ACE2 mRNA found in buccal mucosa smear samples from COVID-19 patients. This has led to the (still very tentative) hypothesis that the cellular response to SARS-CoV-2 infection may be supported by defensive characteristics in order to protect the cells from the viral overload [6,15,82,146,147]. Understanding the precise mechanisms by which cytokines influence COVID-19 infection in the presence of periodontal bacteria and the effect of the infection itself on oral health is still under active study. It is important to continue research to better elucidate these interactions and to develop better strategies for the concurrent management of oral conditions and COVID-19 infection.

## 5. O.C. Pathologies Triggered by COVID-19

SARS-CoV-2 infection in O.C. might directly determine certain clinical features of COVID-19. Several reports postulated that oral lesions could be the first manifestations of the disease since the O.C. tissues are among the SARS-CoV-2 targets [148]. Therefore, the initial disease diagnosis will require an important step performed by dental practitioners, which can then be verified further by patient testing [149,150]. For example, ulcerated gingival lesions might represent the ultimate step of SARS-CoV-2 invasion of oral mucosal cells, mostly through the ACE2/TMPRSS2 pathway, which may subsequently impact oral epithelial cells’ ability to function [27,140]. According to immunohistochemical investigations, T lymphocytic inflammation (CD3) predominates in SARS-CoV-2 focal lymphocytic sialadenitis patients, whereas B lymphocytes (CD20) are comparatively more abundant [151]. Thus, the infection of serous acinar cells in the parotid and submandibular glands, as well as in the O.C., might cause direct cell damage coupled to an inflammatory response within the affected area. This, resulting in clinical consequences, will finally result in xerostomia and salivary gland dysfunction [60,152,153,154].

In COVID-19 subjects, IL-1β, TNF-α, and other proinflammatory cytokines are abundantly found in inflamed gingiva, confirming the local inflammatory condition [12], which can also promote the proliferation of periodontal pathogens within the pockets of the gums. Among them, *Prevotella intermedia*, *Streptococci*, and *Fusobacterium* are, for sure, ideal promoters of acute periodontal conditions [29,43]. As previously noted, a hypothetical vicious cycle might involve the growth of SARS-CoV-2 infection factors in human gingival fibroblasts, which could be triggered by lipopolysaccharides sourced from periodontal pathogens (such as *Porphyromonas gingivalis*) or inflammatory cytokines/mediators (like IL1β and TNFα) [120].

One well-known sign of COVID-19, reported in about 40% of SARS-CoV-2 positive individuals, is the loss of taste, and, interestingly, a strong positive correlation has been reported between SARS-CoV-2 RNA in the saliva and patients’ self-reported “loss of taste” in symptomatic individuals [155,156]. There have been two patients to date who have reported taste abnormalities linked to a high viral load in their saliva and a considerable epithelial (pCK+) cell infection in ACE2-expressing cells [27]. It has also been reported that fungiform papillae taste cells express ACE2 and TMPRSS2 (SARS-CoV-2 entry and transmission factors). Doyle and colleagues also provided evidence of SARS-CoV-2 infection in PLCβ2-positive Type II cells (expressing ACE2), a cell subpopulation of specialized taste receptors present in the taste papillae [157]. SARS-CoV-2 replication was revealed in Type II cells (which have taste receptors for bitter, sweet, and umami stimuli that are G protein-coupled) by in-situ hybridization, strongly highlighting this cell population as a putative portal for viral entry, thus predicting O.C. vulnerabilities to SARS-CoV-2. The same study also showed a persistent disruption of cell turnover in the fungiform papillae taste stem cell layer during infection. Therefore, the clinical manifestation of taste loss might be a consequence of viral cytopathic effects (with local destruction) produced within the papillae taste cells [158]. The decreased frequency of taste loss in people infected with the Omicron variation of SARS-CoV-2 may be explained by functional and molecular differences between the Delta and Omicron forms of the virus. Indeed, the clinical results of taste loss may be explained, at least in part, by variations in the entrance paths of the SARS-CoV-2 variants. The Omicron variation is less fusogenic than the Delta variant and employs a less effective endocytosis entry route (where affinity to ACE2 plays a critical role). However, the Delta virus mostly penetrates through a more effective pathway, which includes host membrane-bound peptidases such as TMPRSS2 [159,160]. The Omicron variant also shows differences at the furin cleavage site within the S1/S2 junction, which can explain reduced fusion capability. It has recently been shown that the Omicron variant possesses a slower replication rate in cells that have an overexpression of TMPRSS2, compared to the Delta variant [160,161]. This observation leads to a hypothetically reduced cytopathic effect, although further study is needed to explore this possibility [162].

## 6. Therapeutic Potential of O.C. against SARS-CoV-2

An early SARS-CoV-2 infection might start in the O.C. Therefore, this anatomical area may be crucial for the transfer of viruses by saliva to the gastrointestinal system or lungs. An analogous mode of infection has been proposed for other microbially-associated disorders, including IBD and pneumonia. Local inflammation and periodontal disease are brought on by viruses that propagate throughout the oral anatomy. As a result, breathing in mouth secretions—which are rich in microorganisms including *P. gingivalis*, *F. nucleatum*, and *P. intermediia*—can contaminate and infect the upper respiratory tract [163]. In the same way, inflammatory cytokines (such as IL-1β and TNF-α) present in inflamed periodontal tissues can potentially enter saliva and be aspirated, potentially triggering inflammation in the lungs [164]. Therefore, proper oral hygiene has been proposed as a means of preventing respiratory infections and subsequent bacterial complications following a viral infection by reducing the occurrence of inter-bacterial spreading between mouth and lungs [165,166,167]. Likewise, containment of oral inflammation might also contribute to preventing SARS-CoV-2 from creating a favorable microenvironment within periodontal pockets, potentially reducing the cellular entry of the virus [91,168]. Bad oral hygiene can also favor virus retention due to the creation of environments that harbor microorganisms (see above) [169,170]. A single clinical case suggests improved oral care resulted in a shorter oral viral load, indicating that rigorous oral care routines can help reduce viral shedding in the O.C. [171].

Treatment with oral antiseptics has been shown to contribute to eliminating SARS-CoV-2 from the O.C. by some preliminary clinical evidence [172,173]. The most effective oral antiseptics against SARS-CoV-2 are, in fact, povidone-iodine, hydrogen peroxide, and cetylpyridinium chloride, which were shown to reduce viral load in COVID-19 patients’ saliva 2-4 h after mouthwashing [173,174]. Additionally, the TMPRSS2 protease activity and spike protein–ACE2 interaction reported for antiseptics may be impacted by the general constituents of toothpastes and mouthwashes. Sodium tetradecene sulfonate, sodium N-lauroyl-N-methyltaurate, sodium N-lauroylsarcosinate, sodium dodecyl sulfate, and copper gluconate, for instance, have been demonstrated to reduce the serine protease activity of TMPRSS2 and the interaction between the receptor-binding domain of spike proteins and ACE2 in vitro assays [175,176]. Altogether, these observations converge on the idea that everyday tools, such as toothpaste and mouthwashes for oral hygiene, might aid in preventing SARS-CoV-2 infection and mitigating the development of COVID-19 complications, thereby potentially improving the disease’s progression [31,156,175]. Natural products, based on cyclodextrins and polyphenols, prevent the entry of the SARS-CoV-2 virus according to the lipid-mediated endocytosis process, reducing the risk of upper respiratory tract infections [177,178]. In conclusion, there is a growing idea that, considering the presence of SARS-CoV-2 in mucosal sites, there is potential in exploring the oral mucosa as a viable target for oral vaccines against SARS-CoV-2, besides being a consistent virus access route. A DNA-based RPS system (recombinant poliovirus sabin-1) from the Sabin-1 viral strain has been explored as a potential delivery system for a COVID-19 vaccine platform [179,180]. This vaccine, based on the RPS-CTP platform, is currently in its early developmental stages, yet it holds promise as a potentially safe and effective oral mucosal prophylactic measure [179,181,182].

## 7. The Role of O.C. in the Diagnosis of COVID-19

It has been described that SARS-CoV-2-positive patients harbor virus particles within acellular and cellular fractions of saliva, making their saliva a valuable tool for diagnosis [183,184]. SARS-CoV-2 RNA saliva-based detection showed a concordance of 96.1% with nasopharyngeal swabs, which serve as the diagnostic gold standard for SARS-CoV-2, with a very high sensitivity and specificity of detection [24,69,185]. Saliva samples from this were self-collected, which might represent a considerable advantage due to its ease of application, particularly in situations where resources in the healthcare system are constrained (when compared to nasopharyngeal swabs) [186,187,188]. Further studies replicated these findings, consistently demonstrating the strong correlation between saliva samples and nasopharyngeal swabs. Additionally, these studies showcased the higher applicability of saliva sampling, indicating its potential superiority, especially in children [189,190]. It is remarkable that SARS-CoV-2 infections were found to be more frequently detected in children through saliva samples compared to nasopharyngeal swabs [191].

To summarize, saliva is nowadays considered a dependable specimen for detecting SARS-CoV-2, offering the advantages of simplicity and non-invasiveness in specimen collection for testing purposes [24,192,193]. Thus, the Food and Drug Administration developed and approved SARS-CoV-2 saliva tests with Emergency Use Authorization [194]. The implementation of tools for collecting saliva in a standardized manner improved the dependability of saliva samples in detecting SARS-CoV-2, and this ameliorated the ability to detect infections pre- and post-symptomatic [195,196].

The detection of anti-SARS-CoV-2 IgA antibodies (detected two days after the beginning of symptoms, an initial and targeted immune response) has gained recent attention during the process of diagnosing COVID-19 [170,197]. Following the appearance of symptoms, these antibodies can remain detectable in saliva for a span of 2 to 3 months [134]. Moreover, since systemic IgG antibodies targeting SARS-CoV-2 may remain detectable, in COVID-19 patients, IgG antibodies against SARS-CoV-2 may persist up to 12 months post-symptom onset. Moreover, sustained IgG antibodies can also be observed in the saliva of asymptomatic individuals [198,199]. The study of mucosal antibody kinetics, which may serve as viable targets for diagnostic techniques to find viral exposure, has received little research to date [21,40,75]. The conversion status of antibody levels (IgA, IgM, and IgG) to SARS-CoV-2 spike and nucleocapsid antigens in saliva can be a valuable indicator for screening people for minimal exposure to the virus [200,201,202].

Salivary amylase blood levels may be a good indicator of a salivary gland SARS-CoV-2 infection. This enzyme enters the bloodstream following the virus-induced death of serous acinar cells, enabling assessment of the extent of damage to the salivary glands in COVID-19 patients [40,202,203,204].

## 8. Clinical Implications

The SARS-CoV-2 virus spreads through saliva, which can therefore be a significant source of virus transmission. Saliva contains high concentrations of viral RNA, with elevated quantities in the first phases of the disease and the presence of infected salivary epithelial cells [205]. The virus can infect salivary gland cells, oral mucosa, and taste buds using entry factors such as ACE2 and TMPRSS2. During simple dental procedures, there are potential risks of virus transmission and implications of the virus for inflammation and pathologies of the O.C. [118,203]. The role of the O.C. is not only limited to the transmission of the virus but also to the diagnosis of the disease [206,207]. Saliva has been recognized as a reliable sample for the detection of SARS-CoV-2, with high sensitivity and specificity comparable to nasopharyngeal swabs [69,208]. Furthermore, saliva is employed to detect specific antibodies against the virus, providing a non-invasive and easy-to-collect diagnostic means [19,167,209]. This study also suggests the importance of oral hygiene to reduce the quantity of the virus in saliva and prevent transmission of the virus [210,211,212]. Furthermore, the potential of mucosa in O.C. as a target for vaccines against SARS-CoV-2 is highlighted. This study therefore provides an in-depth overview of the involvement of the O.C. in the transmission, diagnosis, and possible therapeutic strategies against the SARS-CoV-2 virus [213,214,215].

## 9. Conclusions

Entry and transmission variables for SARS-CoV-2 are largely present in some O.C. structures (keratinized and non-keratinized mucosa and salivary gland epithelia), as shown by a considerable amount of experimental data. Indeed, the infection of SARS-CoV-2 and the viral replication within these structures have been confirmed both at the pre-clinical level (mainly cell culture studies) and in clinical observations. The dynamics of local infection spread, along with the variations in the a-, pre-, and symptomatic stages, are yet to be elucidated. Interestingly, some clinical aspects of COVID-19 disease can be explained through this study of SARS-CoV-2 disease in different cellular populations of the O.C., but additional research is needed to characterize the pathophysiological processes upon SARS-CoV-2 infection. components that consist of cells and substances without cells within saliva have been documented to possess high amounts of viral particles, which can help infective transmission to other individuals (via droplet release) and potentially contribute to the transmission of the virus to both the respiratory and gastrointestinal tracts. These mechanisms, once elucidated, will significantly contribute to a better understanding of COVID-19 physiopathology and clinical significance. Thus, a localized pharmaceutical strategy in the O.C. to combat SARS-CoV-2 might represent a future valuable tool in combating COVID-19, as suggested by promising preliminary clinical observations.

## Data Availability

Data sharing is not applicable.

## Abbreviations

ACEg2	(Angiotensin Converting Enzyme Type 2)
COVID-19	(Corona Virus Disease, 2019)
Iga, IgG, IgM	(Immunoglobulins A, G, and M)
Il1β	(Interleukin 1 beta)
mRNA	(messanger RiboNucleic Acid)
Nrp-1	(Neuropilin-1)
OC	(Oral Cavity)
Pck+	(Pan-Cytokeratin Positive)
Pge2	(Prostaglandin E2)
PGLPS	*(Porphyromonas Gingivalis*-Derived Lipopolysaccharide)
Rps System	(Recombinant Poliovirus Sabin-1)
SARS-CoV-2	(Severe Acute Respiratory Syndrome Coronavirus 2)
TMPRSS2	(Transmembrane Serine Protease 2)
Tnfα	(Tumor necrosis factor)
